# Implications of global distributive justice principles for implementation of the Kunming‐Montreal Global Biodiversity Framework

**DOI:** 10.1111/cobi.70167

**Published:** 2025-10-30

**Authors:** Ina Lehmann, Marcel Kok, Roos Immerzeel, Alexandra Marques

**Affiliations:** ^1^ Institute for Environmental Studies (IVM) Vrije Universiteit Amsterdam Amsterdam The Netherlands; ^2^ PBL Netherlands Environmental Assessment Agency The Hague The Netherlands

**Keywords:** Convention on Biological Diversity, 2030 targets, ability to pay, beneficiary pays, contributor pays, capacidad de pago, Convenio sobre la Diversidad Biológica, objetivos 2030, pagos del beneficiario, pagos del contribuyente, 《生物多样性公约》, 2030年目标, 贡献者付费, 受益者付费, 支付能力

## Abstract

In the era of the sixth mass extinction, reversing global biodiversity loss is of vital importance for life on Earth. In 2022, parties to the Convention on Biological Diversity (CBD) adopted the Kunming‐Montreal Global Biodiversity Framework (GBF), a strategic plan with 23 action‐oriented targets to be achieved by 2030. However, biodiversity action carries direct and indirect costs that are unevenly distributed globally. Moreover, parties to the CBD may differ relative to various aspects of bearing these costs. Although the GBF implicitly acknowledges its parties’ common but different responsibilities for its implementation, what this means in practice is left open. We suggested a distributive justice framework to guide global sharing of the costs of biodiversity action, with a focus on specific GBF targets. We combined the contributor pays, beneficiary pays, and ability to pay principles from the normative‐philosophical justice literature together with empirical information on trends in biodiversity degradation, benefits from resource exploitation, and livelihood levels in different countries to develop a distinct and comprehensive account of distributive justice in a global biodiversity policy context and to specify implications for target implementation. Our framework suggests that high‐income countries should provide substantial financial resources for the implementation of GBF targets domestically and internationally. Moreover, these countries have a particularly high moral obligation to take action to reduce pressure on biodiversity—for instance, by reducing pollution or changing consumption patterns. Recent institutional innovations related to the funding, planning, monitoring, reporting, and review structures of the GBF hold promise for its just implementation, which ultimately depends on parties’ political will.

## Abstract (es)

Consecuencias de los principios de justicia global distributiva en la implementación del Marco Mundial de Biodiversidad Kunming‐Montreal

**Resumen:**

En la era de la sexta extinción masiva, revertir la pérdida de biodiversidad global es de vital importancia para la vida en la Tierra. En 2022, las partes del Convenio sobre la Diversidad Biológica adoptaron el Marco Global de Biodiversidad de Kunming‐Montreal (MGB), un plan estratégico con 23 objetivos orientados a la acción que deben alcanzarse para 2030. Sin embargo, las medidas en favor de la biodiversidad conllevan gastos directos e indirectos que se distribuyen de forma desigual a nivel mundial. Además, las partes del CDB pueden enfrentarse a circunstancias diferentes en relación con diversos aspectos de sobrellevar estos costes. Si bien el MGB reconoce implícitamente las responsabilidades comunes pero diferentes de sus partes en cuanto a su aplicación, no queda claro lo que esto significa en la práctica. Planteamos un marco de justicia distributiva para orientar el reparto mundial de los costes de las medidas en favor de la biodiversidad centrado en objetivos específicos. Combinamos los principios de «quien contribuye paga», «quien se beneficia paga» y «capacidad de pago» de la literatura normativa‐filosófica sobre justicia con información empírica sobre las tendencias en la degradación de la biodiversidad, los beneficios de la explotación de los recursos y los niveles de subsistencia en diferentes países para desarrollar una visión clara y completa de la justicia distributiva en el contexto de las políticas globales de biodiversidad y especificar las implicaciones para la implementación de los objetivos. Nuestro marco sugiere que los países de altos ingresos deberían proporcionar recursos financieros sustanciales para la implementación de los objetivos del MGB a nivel nacional e internacional. Además, estos países tienen una obligación moral muy elevada de tomar medidas para reducir la presión sobre la biodiversidad, por ejemplo, la reducción de la contaminación o cambios en los patrones de consumo. Las recientes innovaciones institucionales relacionadas con las estructuras de financiación, planificación, seguimiento, presentación de informes y revisión del MGB son prometedoras para su implementación justa, que en última instancia depende de la voluntad política de las partes.

## 全球分配公正原则对实施《昆明‐蒙特利尔全球生物多样性框架》的影响

**【摘要】** 在第六次生物大灭绝的时代, 扭转全球生物多样性丧失对地球上的生命至关重要。2022年, 《生物多样性公约》缔约方通过了《昆明‐蒙特利尔全球生物多样性框架》(GBF), 这是一项战略计划, 包含23个需在2030年前实现的以行动为导向的目标。然而, 生物多样性行动需要付出直接与间接成本, 而这些成本在全球的分布并不均衡。此外, 《生物多样性公约》缔约方在承担这些成本的各个方面可能面临不同情况。虽然GBF隐含地承认了缔约方对其实施负有共同但不一致的责任, 但这在实践中意味着什么却尚无定论。我们提出了一个分配公正框架, 以指导全球各国分担生物多样性行动的成本, 重点关注具体的目标。我们将规范‐哲学正义研究中的“贡献者付费”、“受益者付费”和“支付能力”原则与不同国家生物多样性丧失趋势、资源开发收益和生计水平的经验信息相结合, 对全球生物多样性政策背景下的分配正义进行了独特且全面的阐述, 并明确了实施目标的意义。我们的框架表明, 高收入国家应为在国内和国际上实施GBF目标提供大量财政资源。此外, 这些国家有非常高的道德义务来采取行动, 以减少对生物多样性的压力, 例如通过减少污染或改变消费模式。最近与GBF的资金、规划、监测、报告和审查结构相关的制度创新为其公正实施带来了希望, 而其成功与否最终取决于各个缔约方的政治意愿。**【翻译:胡怡思;审校:聂永刚】**

## 1 INTRODUCTION

Nature and biodiversity provide many benefits to people, often referred to as nature's contributions to people (IPBES, 2022), including essential goods, such as food and fiber, and services, such as water purification and recreation. Nature also shapes human cultures, which underscores the relational value of nature. For example, food provides not only needed calories and nutrients but also enjoyment and is at the heart of cultural identities. Yet, biodiversity and ecosystems are threatened worldwide; species loss rates are 10,000 times higher than natural background rates (Ceballos et al., 2015). According to IUCN (2024), over 44,000 species are threatened with extinction. This underlines the intrinsic value of biodiversity (IPBES, 2022) and threatens human livelihoods. The negative effects of biodiversity loss on livelihoods are already being felt. For instance, in some places, the loss of pollinating insects is affecting food security (IPBES, 2016). Ninety percent of fish populations were within biologically sustainable limits in 1974. This number declined to 67% in 2015, which means important protein sources for many people have been lost (FAO, 2018). As biodiversity decline continues, such negative effects are likely to worsen (IPBES, 2019).

In response to these developments, the Convention on Biological Diversity (CBD) was adopted in 1992 to provide guidance to its 196 member states (https://www.cbd.int/information/parties.shtml) on 3 objectives: conserving biodiversity, sustainably using its components, and equitably sharing the benefits from the utilization of genetic resources (UN, 1992). After years of failing to halt biodiversity loss, at its 15th Conference of the Parties (COP) the parties adopted the Kunming‐Montreal Global Biodiversity Framework (GBF) as strategic guidance for setting the world on a path to “living in harmony with nature” (UN, 2022a). It promotes actions to mitigate threats to biodiversity and improve its state through 4 broad, long‐term goals for 2050 and 23 action‐oriented targets for 2030 that may require significant changes in current practices in the use of nature and natural resources. For example, target 3 asks for the protection of 30% of global land and sea areas, and target 7 calls for the reduction of pollution risks globally to levels that are not harmful to biodiversity. Yet, apart from a generic acknowledgment that countries have different capacities to implement the GBF and that some countries may need international support (articles 7 and 15), the GBF does not specify to what extent and how individual countries are to implement its global goals and targets.

How countries, with their varying circumstances and capacities, should respond to GBF requirements raises important questions of justice. Think, for example, of the direct costs of establishing and maintaining a protected area, including direct costs, such as the salaries of park managers, and opportunity costs, including limiting resource extraction. Who should share in these costs, the countries in which direct threats to ecologically valuable areas arise or the wider international community that benefits from biodiversity conservation? Or, think of the short‐term output and income losses for farmers requested to reduce fertilizer use and the income losses for fishers whose fishing quotas are lowered. Should social benefit schemes be in place to compensate for their income losses? If so, who should pay for such compensation schemes? On a global scale, international trade is a major driver of land degradation and biodiversity loss (Marques et al., 2019). To what extent are importing and exporting countries of natural resources responsible for this and what should their share of the costs of landscape restoration be?

The GBF does not answer such questions. Yet, answers are needed because intrinsically, social relations associated with conservation should be organized justly, and instrumentally, perceived injustices can trigger opposition to biodiversity conservation (Martin, 2017). We devised an empirically informed distributive justice framework to provide normative guidance for the globally just implementation of the GBF. The framework can be used as a basis for assessing to what extent the GBF is being implemented justly. More specifically, we sought to help global environmental justice scholarship extend beyond climate change to biodiversity policy.

Since Henry Shue's (1999) seminal essay, “Global Environment and International Inequality,” normative accounts of what constitutes globally just sharing of the remaining emissions budget have been advanced relative to long‐term temperature goals (e.g., Caney, 2005, 2006, 2010; Holz et al., 2018; Moellendorf, 2014; Page, 2012, 2016). More recently, debates about planetary justice (Biermann & Kalfagianni, 2020; Kashwan et al., 2020) or safe and just Earth System boundaries (Gupta et al., 2023; Rockström et al., 2023) promote a holistic view of justices and injustices across diverse, interconnected global challenges. Discussions of justice of global biodiversity governance are slowly gaining momentum (Pickering et al., 2022; Scobie, 2021; Stark et al., 2022). Pettinotti et al. (2024) suggest quantifying countries’ fair share of global biodiversity finance without normatively justifying some key decisions informing their calculations. In normative‐philosophical justice scholarship, Armstrong (2017, 2019, 2024) has promulgated important advances while maintaining a strong focus on conservation. Thereby, Armstrong does not cover the breadth of distributive justice concerns related to biodiversity and nature's contributions to people, including direct and indirect drivers of biodiversity loss, and we aimed to shed a distributive justice light on biodiversity governance more broadly. Moreover, even if Armstrong goes far in grounding philosophical analyses in empirical examples, he does not offer specific policy guidance. Inspired by Armstrong's reasoning, we also sought to add relevant information about the global policy context and deepen empirical insights into the costs and benefits associated with biodiversity loss in the hope that our framework will offer insights into real‐world patterns of responsibility. We also hope that it will provide normative guidance with political and practical traction for CBD parties as they implement their 2030 commitments.

We based our work on a few fundamental premises. We assumed that reversing biodiversity loss is itself a demand of justice for current and future human generations, whose physical and mental well‐being depends on nature, and for nonhuman species. We also posited that nature has intrinsic value, and this value strengthens the imperative to reverse biodiversity loss (Armstrong, 2024). However, we focused on justice between humans as organized in states because these are the politically relevant actors for the just implementation of the GBF. We therefore did not consider ethical discussions on whether the value of nature translates into rights of nature or demands of justice (Kalfagianni et al., 2024). Instead, we considered how the estimated costs of reversing biodiversity decline by 2030, which range from US$722 to US$967 billion/year (Deutz et al., 2020), and which are likely on the high end of the spectrum (Bromley, 2024), should be allocated justly among CBD parties. Such a discussion is at the heart of the notion of distributive justice, which is conceptually distinct from notions of procedural justice (who has a voice in decision‐making) and recognitional justice (whose values and beliefs are acknowledged) (Schlosberg, 2007). Distributive patterns may be influenced by procedure and recognition (Schlosberg, 2007). However, we focused on distributive justice among CBD parties because allocating responsibility for implementation of GBF targets requires an understanding of justifiable demands on national efforts and because controversies around the costs of biodiversity action dominate the international political debate about GBF implementation (Greenfield, 2022). The GBF targets are politically negotiated and potentially not sufficiently ambitious to reverse biodiversity loss (Kok et al., 2024). However, because they will guide action until 2030, we took them for granted and considered how the international community could justly implement them.

## 2 THE CBD AND GBF

The CBD has a reputation as a particularly strong sustainable‐development‐oriented multilateral environmental agreement (Le Prestre, 2002; Prip et al., 2010). It implicitly incorporates the principle of common but differentiated responsibilities (CBDRs) for allocating the costs of environmental action as agreed upon by the international community at the Rio Earth summit in 1992 (UN, 1992), meaning that mitigating global environmental change is a globally shared responsibility, but not all states need to take the same level of action. In this spirit, several CBD articles (e.g., 8, 10, 11) emphasize the links between biodiversity conservation and social development and highlight the role of stakeholders. Moreover, the CBD (articles 18, 20, 21) foresees for international cooperation and support for developing countries to implement its requirements.

However, the CBD provides only the overall structure for international cooperation and does not quantify goals and targets or specify who is responsible for undertaking what action (UN, 1992). The COP's decisions have therefore been instrumental in specifying guidance in fields as diverse as protected areas, agricultural biodiversity, forest biodiversity, sustainable use of biodiversity, biodiversity finance, and many more (https://www.cbd.int/decisions). Moreover, several COP decisions have strengthened the links between biodiversity conservation and sustainable development and highlight the role of stakeholders, such that concerns with justice can rightfully be considered central to the CBD.

The 23 action‐oriented targets of the GBF for 2030 specify expectations from CBD parties more concretely than previous COP decisions and strategic action frameworks, including the GBF's predecessor, the Aichi Targets (UN, 2010). The GBF's overarching aim is “to catalyze, enable and galvanize urgent and transformative action … to halt and reverse biodiversity loss” and to support the CBD's overall commitment to justice and sustainable development (UN, 2022a). The term *equity*, a notoriously vague word in international environmental law, is used several times, for example, in calls for equitable governance of protected areas (target 3), equitable reduction of the global footprint of consumption (target 16), and equitable reduction of biodiversity‐harming subsidies (target 18). In GBF Article 7d, the CBDR principle is recognized when it is noted that although the goals and targets of the GBF are “global in nature,” each party will contribute to them in accordance with their own circumstances and capabilities, and GBF Article 7f recognizes the right to development. Article 15 notes that implementation of the GBF will require the availability of financial resources for parties in need of extra support, and article 18 acknowledges that poorer countries will need implementation support, including capacity‐building, technical, and financial support. Finally, target 19 calls for biodiversity‐related international financial transfers from developed to developing countries (according to UN categories) of at least US$20 billion per year by 2025 and at least US$30 billion per year by 2030. The GBF thus provides some guardrails for global distributive justice, but, because they are outcomes of political negotiations, these still do not provide concrete guidance for the just implementation of the GBF.

Taking a step back from power‐ and interest‐laden negotiation processes, we drew on insights from environmental justice philosophy to suggest more specific guidance for sharing the costs associated with the implementation of the GBF and its different targets. To make this task manageable while sufficiently concrete, we limited our analyses to selected targets with direct biophysical or ecological objectives (thus not targets related to information sharing, capacity building, etc.). We also excluded the GBF's approach to the fair and equitable sharing of the benefits from the utilization of genetic resources. This raises separate ethical, legal, and political questions around intellectual property rights that we could not address here (e.g., Morgera, 2024). We use the shortened titles of selected targets and provide a full list of all GBF targets in Appendix S1 and the full text of the selected targets in Appendix S2.

First, we zoomed in on area‐based targets, that is, targets that address action for a specified amount of land or sea surface. More concretely, we considered the distributive justice requirements for targets 2 (restore 30% of all degraded ecosystems) and 3 (conserve 30% of land, water, and seas). Second, we examined a target related to the reduction of pressures, namely target 7 (reduce pollution to levels that are not harmful to biodiversity). Third, we covered 2 targets addressing benefits for people: targets 10 (enhance biodiversity and sustainability in agriculture, aquaculture, fisheries, and forestry) and 11 (restore, maintain, and enhance nature's contributions to people). Finally, in relation to targets addressing indirect drivers of biodiversity loss and degradation, we reflected on a specific part of target 16 (enable sustainable consumption choices to reduce waste and overconsumption): equitable reduction of the global footprint of consumption. All these targets delineate global expectations, but they do not specify what is required at a country level (i.e., how benefits and burdens of implementing the targets should fall on different countries) or according to which specific principles implementation support should be flowing from higher to lower income countries. This presents the gap that our subsequent analysis seeks to address.

## 3 PRINCIPLES OF DISTRIBUTIVE JUSTICE FOR BIODIVERSITY GOVERNANCE AND THEIR IMPLICATIONS FOR GBF TARGETS

Because many species and ecosystems are highly place specific, biodiversity recovery must occur in situ, where threats to biodiversity manifest (Armstrong, 2019). Moreover, because all countries experience biodiversity loss and degradation (IPBES, 2019), all governments are morally obliged to undertake and support measures for the conservation, restoration, and sustainable use of biodiversity where species and ecosystems on their territories are under threat. A generic determination of per‐country shares of global biodiversity action according to economic power, comparable to emission reduction shares under the UN Climate Convention's Kyoto Protocol (UN, 1997), is thus not morally appropriate. In contrast, international sharing of the opportunity costs of local or national biodiversity action in line with the CBDR principle will often mean that some countries have to provide technical and financial support for action in other countries. Determining how much of these costs should be nationally versus internationally borne, and if internationally by which states, is central to our analyses. We built our argument on 3 general, complementary, and widely recognized global environmental justice principles and considered their implications for our selected GBF targets (Armstrong, 2017, 2019, 2024). Although data gaps prevent precise distributive recommendations, existing trends allowed us to offer general normative guidance for the just implementation of these targets.

### 3.1 Contributor pays

The contributor pays principle is known from climate justice debates as the polluter pays principle (Shue, 1999; Singer, 2002). In its most straightforward formulation, it is based on the intuitively appealing principle *you broke it, you fix it*, meaning that whoever causes a problem ought to accept responsibility for eliminating its adverse consequences (Singer, 2002). In a biodiversity context, according to this principle, actors should bear the costs of correcting environmental harm in proportion to their own contribution to it (Page, 2016), which may include the opportunity costs of reducing threats to ecosystems or species (Armstrong, 2024). Assigning responsibility for causing a problem is a pressing consideration because the role of anthropogenic factors in driving biodiversity loss, including direct drivers such as resource overexploitation, land‐use change, pollution, and climate change and indirect drivers, such as production and consumption patterns, is well established (IPBES, 2019). Consumption patterns are particularly important for determining where responsibility ultimately falls. Although direct biodiversity harm takes place where natural resources are overexploited or ecosystems are polluted, this often happens through teleconnected systems of international trade to meet the demands of consumers elsewhere (Marques et al., 2019); thus, places of consumption are where the ultimate motivation for the exploitation of nature originates.

Although establishing individual countries’ responsibility for biodiversity loss and degradation is not straightforward due to the many actors and processes involved, biodiversity footprint calculations offer a useful proxy. They draw an aggregate picture of the per capita level of consumption‐based biodiversity losses attributable to each country. In a widely recognized study of 45 countries and world regions, Wilting et al. (2017) showed, for instance, that every country overuses biodiversity to some extent, even though the footprint varies significantly among countries and regions. As a general trend, the per capita biodiversity footprint increases as the economic wealth of a country increases. North America, Europe, and Latin America (in this order) have above‐average biodiversity footprints. Conversely, the average per capita biodiversity footprint of Asian and African countries is below the global per capita average. In some countries, such as India, it is substantially below the global average.

Biodiversity footprint takes teleconnections into account and is calculated as the combined domestic and foreign pressure, with the latter reflecting the extent to which countries contribute to biodiversity loss abroad through imports (Wilting et al., 2017). Biodiversity footprint studies and other research on international trade show that high‐income countries of the Global North significantly drive biodiversity loss in other parts of the world through the consumption of internationally traded agricultural food commodities and deforestation related to this consumption (Cabernard et al., 2024). On average, the share of imported pressure on biodiversity increases as the economic wealth of a country or region increases, meaning that Europe (Western European countries in particular), the United States, and Japan import far more biodiversity loss per capita than they export (Wiebe & Wilcove, 2025; Wilting et al., 2017). More concretely, roughly 50% of European Union's pressures on land use and biodiversity happen outside the European Union (Bruckner et al., 2023). In contrast, biodiversity losses due to imports and exports to and from Asia are roughly in balance, whereas South America and Africa primarily export biodiversity losses to other regions, such as Europe and North America (Wilting et al., 2017). Approximately half of African exported losses from environmental pressures are the result of European consumption (Wilting et al., 2017), and 75% of land‐use‐change impacts in Africa are driven by its overall exports, with key destinations of increased exports depending on the commodity (Cabernard et al., 2024).

For some emerging economies, a more differentiated and partly inconclusive picture arises. For instance, China is a major exporter of biodiversity loss to other regions, such as Europe and North America (Wilting et al., 2017). In Indonesia and Brazil, domestic contributions are key drivers of biodiversity loss through deforestation according to one study (Wiebe & Wilcove, 2025). Another study showed that beef exports, mainly to China, the Middle East, and Europe, account for 70% of Brazil's net land‐use change impacts on biodiversity, and Indian demand is among the key drivers of increasing oilseed exports from Indonesia, thereby further driving deforestation (Cabernard et al., 2024). Still, India's per capita biodiversity footprint remains significantly below the global average (Wilting et al., 2017).

What do these patterns and trends mean for the application of the contributor pays principle for the just implementation of the GBF? Because no country has zero biodiversity footprint, their common responsibility requires all countries to take domestic action and bear direct and opportunity costs of implementing GBF targets—for instance, by setting aside protected areas from human use, restoring degraded biodiversity, or making current production and consumption patterns more sustainable. Given the vast differences in countries’ contributions to the problem, however, their responsibility is not only common but also different. First, countries that are particularly strongly driving biodiversity loss and degradation through pollution (target 7), unsustainable use (target 10), or general consumption patterns (target 16) ought to take particularly strong action to transform relevant economic sectors. In emerging economies, such as Indonesia or Brazil, this may largely apply domestically. High‐income countries with high biodiversity footprints and imported biodiversity degradation are also under particular moral obligation to stop externalizing pollution and overuse of biological resources (such as overfishing or agricultural land use) in other, often lower income, countries Second, in line with the teleconnection patterns outlined above, this primarily means that high‐income European and other Global North countries, as well as potentially some emerging economies, such as China, should share in the costs of biodiversity action in low‐income countries, in particular the costs of reaching the targets of conserving existing threatened biodiversity and people's benefits from it, both locally and globally (targets 3 and 11), and restoring degraded biodiversity and associated human benefits (targets 2 and 11). This is the part of their obligations that they can only fulfil through technical and financial assistance given that biodiversity action has to primarily take place in situ in affected areas.

However, on its own, the contributor pays principle runs into problems, as causal responsibility for biodiversity loss and degradation does not always translate into moral responsibility. First, the contributor pays principle has little traction for allocating costs of correcting past biodiversity loss. If one considers humans, including when they are collectively organized in states, as autonomous actors who should take responsibility for the consequences of their own actions, it is morally questionable to hold the present generation accountable for the wrongdoings of their ancestors on which they had no influence (Armstrong, 2017, 2024).

This does not ignore the fact that biodiversity loss increased tremendously after 1945 (Millennium Ecosystem Assessment, 2005) or that with accelerating globalization, countries of the Global North increasingly externalized biodiversity loss. For instance, since 1961, crops for export have expanded more than twice as fast as crops for domestic consumption (Levers & Müller et al., 2019). The currently living thus bear most of the causal and moral responsibility for biodiversity loss and degradation because it happened during their lifetime. This should, however, not conceal significant historical biodiversity loss and degradation, which continue to contribute to less resilient ecosystems today. Early biodiversity threats included the decimation or elimination of species through hunting—some whale species are telling examples (Andresen, 2002). The globally dominant historical driver of biodiversity degradation, however, was deforestation, notably for the expansion of agriculture and the often‐unsustainable use of forests for raw material and energy for economic development, particularly during the industrial revolution (FAO, 2012). Externalization of biodiversity harm took off with industrialization in the 19th century when European and North American resource demands accelerated. Colonialism expanded the plantation economy, which destroyed entire landscapes as large quantities of sugar, tobacco, coffee, cocoa, and other agricultural resources were exported to Europe and became bulk commodities (Radkau, 2000). Particularly in Asia, colonial timber exploitation also strongly drove ecological harm displacement (FAO, 2012). The rich countries have thus historically contributed to serious biodiversity harm both at home and abroad for which their current citizens should not be held responsible as contributors.

A second limitation of the contributor pays principle arises in association with actors who have no meaningful alternative to natural resource overexploitation and therefore cannot reasonably be held accountable for their actions (Armstrong, 2024, pp. 62–64). In particular, this raises the question of what sacrifices people and communities, in particular poor people and communities, may be asked to make for the global benefit of biodiversity conservation. If the aim of distributive justice is to factor in people's living conditions, at the very least, the contributor pays principle should not turn into a mechanism that furthers poverty. Further discussion is thus needed on the economic preconditions for bearing a share of the costs of reversing biodiversity loss.

Another potential limitation of the contributor pays principle discussed in the climate justice debate is excusable ignorance because there is no moral foundation for holding actors responsible if they cannot be expected to understand the harmful consequences of their actions (Caney, 2006). Although excusable ignorance may apply, for example, when awareness of the harmful effects of greenhouse gas emissions is not sufficiently widespread, the potential role of excusable ignorance in the biodiversity crisis is much smaller. Certain practices or inputs may sometimes be deployed in the genuine but false belief that they are harmless. By and large, however, overexploitation of natural resources, pollution, and intensification of resource use have been widely known as drivers of biodiversity loss for decades, if not centuries (Guha, 2000; Radkau, 2011).

However, even if one accepts only the first 2 limitations of the contributor pays principle, our initial premise that reversing biodiversity loss is itself a demand of justice does not allow letting the costs that the contributor pays principle cannot cover to go unaccounted for because that would open the door to inaction. Rather, other distributive justice principles are needed to complement and fill the gaps left open by the contributor pays principle.

### 3.2 Beneficiary pays

The first complementary principle fills some gaps left open by concerns over historical biodiversity degradation. It shifts attention from contributors to beneficiaries, hence its name beneficiary pays principle. We disagree with Armstrong's (2024) assignment of a marginal role to the beneficiary pays principle to specify the kind of wrong committed by actors who do not fulfil their obligation of paying for biodiversity action. Considering that the contributor pays principle leaves open where some costs of biodiversity action should fall, in contrast, the beneficiary pays principle is a useful first step toward filling some of these gaps. Our key argument for this principle is centered around a motivation to avoid unjust benefits from harmful action. According to this argument, the living have inherited responsibility for previous biodiversity harm from their ancestors because the benefits they have thereby derived are principally undue (Caney, 2010; Page, 2012). It would amount to freeriding if people were allowed to unrestrictedly benefit from environmental harms and would socialize the costs but not the benefits from biodiversity‐harming activities (Gosseries, 2004). Rather, people should bear additional costs of biodiversity action when they have gained from environmentally harmful past activities (this argument has been developed in the climate change context for instance by Caney [2005, 2006] and Page [2012]).

With a view to just GBF implementation, the empirical question then is which countries have benefited from past biodiversity overexploitation and how much. By and large, the overuse of biodiversity and natural resources poses a long‐term threat to lasting wealth, rather than contributing to it, because it depletes the natural foundations of livelihoods and well‐being (Otero et al., 2020). However, some historical overexploitation contributed to the economic development of today's high‐income countries. The economic growth associated with industrialization in Europe was significantly fueled by large‐scale exploitation of natural resources in the colonies. The British (followed by the Dutch, Portuguese, French, Belgians, and Germans) were world leaders in deforestation, notably decimating forests in the northeastern United States, southern Africa, and India (Guha, 2000). Timber imported from India, for instance, was used by the British for shipbuilding, iron smelting, and tanning. Moreover, the British cleared Indian forests for agriculture and for building railways across the subcontinent to support colonial trade (Guha, 1983). It is partly the wealth created through these environmentally destructive processes that now allows high‐income countries to replace their own lack of natural resources with those from low‐income countries, as in the case of timber or fish (FAO, 2012; Millennium Ecosystem Assessment, 2005; Radkau, 2000). In addition to infrastructure developments, the push of the commodities frontier in the settler colonies from the 1850s onward enabled increasing amounts of wheat and meat being shipped first to Britain and with some delay to other European countries, such as Germany, France, and Italy. This not only contributed significantly to these countries’ land footprints but also helped secure cheap food for the masses of industrial workers, which was instrumental to economic specialization and development and the competitive advantages that persist today (Krausmann & Langthaler, 2019).

Historical evidence thus shows that certain past practices, associated largely with the exploitation of natural commodities in the European colonies, contributed to laying the foundations for the current wealth of many European countries. Despite its different starting point, the beneficiary pays principle thus supports the argument of the contributor pays principle that it is the responsibility of the high‐income European countries to financially and technically support biodiversity action across all GBF targets in the Global South, including middle‐income countries, such as India, where resources for the industrial revolution in Europe were extracted. Applying these considerations to our selected GBF targets moreover supports the call to avoid accumulating further short‐term economic benefits while harming biodiversity globally. Again, this reinforces the normative call to rich countries in particular to reduce their consumption footprint (target 16) and all polluting and intensifying practices (targets 7 and 10).

### 3.3 Meeting human needs and ability to pay

Because not all past biodiversity harm was translated into long‐lasting benefits, the beneficiary pays principle still leaves some costs of historical overuse unaccounted for. We suggest that this remainder be covered by the high‐income countries. Likewise, these countries should cover the costs of biodiversity action that would be too much of a burden for low‐income countries to address the second limitation of the contributor pays principle related to poverty. After starting from contributions to, and benefits from, harm as guiding normative principles, it may initially appear unduly simplifying to allocate all remaining costs and costs that may seem too burdensome for low‐income countries to high‐income countries. Yet, once other principles are exhausted, we consider it appropriate to side with the economically weaker (Caney, 2010; Vanderheiden, 2011), thus making this a strong case for differentiated responsibilities. This argument is informed by the widely shared conviction that the essence of human existence must be at the core of distributive justice and must always be guaranteed (Griffin, 2008; Odera Oruka, 1997). Hence, the possibility for countries, and thereby their residents, to meet a specific level of livelihood has to be exempted from considerations of responsibility for biodiversity harm and potential burden sharing.

This has 2 implications. First, actors causally responsible for biodiversity harm should not be held morally responsible according to a contributor pays principle if they had no option to act differently without facing unreasonable costs. That is, one cannot morally expect people to forego key human needs for the sake of maintaining biodiversity, and if these needs require them to overuse natural resources locally, they should not be punished further by holding them responsible for the costs of conservation (Armstrong, 2024). Industrialized production and consumption patterns, notably in the Global North, clearly are the main drivers of biodiversity loss and degradation (IPBES, 2019). However, situations of local overuse are not uncommon in many countries of the Global South when rural people have no choice but to overexploit natural resources for their daily needs, thereby often tapping into a downward spiral of increasing poverty and decreasing local environmental quality (CBD, 2010; Millennium Ecosystem Assessment, 2005). Accordingly, the poverty of large parts of the population of poorer countries at least partially excuses their natural resource overuse and thus lowers their moral obligation to share in the costs of biodiversity action.

Second, the moral requirement to guarantee people's livelihoods implies that nobody should be impoverished or further impoverished by environmental action, such as use restrictions or any other measures that reduce the availability of natural goods and services for those who urgently depend on them. In environmental justice philosophy, this idea is widely known as the ability to pay principle. In the words of Shue (1980, p. 18), this principle epitomizes a “morality of depth (…) the line beneath which no one is to be allowed to sink.” Where actions to reverse biodiversity loss will foreseeably result in the temporary or permanent loss of basic livelihood opportunities, such as access to vital natural resources or widely affordable biologically based products, some form of compensation is morally required to prevent the burden from falling on the poor. If the home countries of affected communities are unable to provide compensation themselves without, for example, cutting essential services, a responsibility to financially support compensation shifts to high‐income countries that can afford this without major burdens on their own populations (this broadly follows an argument in relation to climate change by Moellendorf [2014]).

If the 2 related principles of meeting human needs and ability to pay bolster the differentiated responsibility of the higher income countries to support biodiversity action in low‐income countries, the question still is what the threshold should be for largely exempting low‐income countries from bearing the costs of biodiversity action. In a world of plenty, it can hardly be considered just if people merely have the means to barely survive. Rather, the means to a decent livelihood may determine such a threshold, including for instance the means to be well nourished and to have a good level of health and access to good shelter (Sen, 1985, 1999). This may also imply more than having enough calories per day to maintain a normal weight and can include access to a balanced and healthy diet and a level of food sovereignty, meaning that people have a choice of groceries that reflect customary understandings of good food (Via Campesina, 2002). Biodiversity action more generally should thus protect and promote access to nature's contributions to people to an extent that allows meeting the requirements of a decent living. Even though country income levels are an insufficient indicator of decent livelihoods, there is at least some correlation between the 2, and aggregate income frequently reflects countries’ capacity to pay for environmental measures. Taking gross domestic product (GDP) as a practical guidance, the above considerations mean that not only the low‐income countries according to UN or World Bank classification should be largely exempted from costs of biodiversity action due to their very low GDP levels. To draw a line of decent livelihoods, the threshold for largely exempting countries should rather be drawn somewhere in the group of lower middle‐income countries (this argument broadly draws on Kartha et al. [2009]).

Rather than setting a fixed absolute threshold, however, a case can also be made for a more egalitarian approach. If one sees biodiversity governance as part of broader global justice questions and if one aims not only for a decent level of living around the world but also for equalizing living conditions globally, one could consider some indexing that factors in countries’ GDP and human development levels in determining their ability to pay for biodiversity action (Armstrong, 2017). Thus, the economically relatively better off a country is, the more it can be morally required to contribute to biodiversity action and funding before the ability to pay principle threshold is reached (Caney, 2010; Shue, 1999). Although ability to pay baselines largely exempt the low‐income and partly lower middle‐income countries from bearing costs of biodiversity action, an egalitarian baseline would allocate even more global funding responsibility to upper‐middle‐ and high‐income countries.

Looking more closely into the selected GBF targets, the question of how far their domestic implementation threatens to undermine livelihoods in low‐income countries arises. Specific evidence is available with regard to GBF target 3, which requires setting biodiversity aside from the threats of human use by expanding protected area coverage to 30% of land and sea globally. To meet target 3, lower income countries would face higher increases in the amount of land and sea under conservation and, accordingly, would face relatively higher opportunity and establishment costs than high‐income countries. The budget increase for protected areas (given current levels of international aid) would amount to 0.32% of GDP for low‐income countries—more than 3 times the increase for high‐income countries (Waldron et al., 2022). Some of the most species‐rich areas moreover occur in the tropics where poverty rates are high; “heavy conservation burdens in these regions could therefore exacerbate poverty and social injustice” (Shen et al., 2023, p. 550). This is the case because many areas identified as priority conservation areas and protected areas are in human‐modified landscapes.

To achieve protection of global priority areas for conservation, many low‐income countries would also face conservation challenges disproportionate to their economic capacities. This can be expected to intensify trade‐offs between future expansion of protected areas and human development (Shen et al., 2023). Similarly, tremendous pressure on forest cover in middle‐income countries, such as Mexico, drives up their costs of biodiversity protection (Waldron et al., 2022). Eventually, protected area personnel and ranger numbers globally are only at about 36% of what is needed to effectively manage existing protected areas. These numbers are particularly low in the tropics, where only 22.4% of protected areas have adequate staffing and budgets. Extrapolating these numbers to what will be needed to meet target 3 translates into a requirement of additional 3 million park managers and 1.5 million rangers globally, with a large proportion of them in low‐income countries (Appleton et al., 2022). Again, this shows that action is required most urgently where the means may be most deficient, thus reinforcing the argument for international cooperation and support mechanisms.

Similar estimates of the costs of implementing the other GBF targets are not available. Of relevance for target 2, however, restoration is much costlier and less successful than the preservation of species and original habitat (Mori & Isbell, 2023). Moreover, degraded ecosystems behave in complex and nonlinear ways, making it unpredictable at what point restoration will become prohibitively expensive (Mori & Isbell, 2023). Again, implementing target 2 will likely challenge the lower income countries in particular. Similar reasoning applies to the other targets as changes in production patterns and measures to enhance nature's contributions to people, as addressed in target 11, can generally be expected to come with opportunity, investment, management, and monitoring costs, which entail a higher burden on poorer countries. Target 16 and its requirement of halving the global consumption footprint is an exception because it is primarily aimed at high‐income countries and thus represents little extra burden for poorer countries. This, however, does not change the overall conclusion that poorer countries’ limited ability to pay for some of the most prominent GBF targets morally requires substantial international support from the richer countries.

Based on the premise that reversing biodiversity loss itself is a demand of justice for current and future generations, our main concern has been to identify guidance for the just sharing of the direct and opportunity costs of GBF implementation, thereby paying attention to its different facets of conservation, sustainable use, production and consumption patterns, and so forth. To this end, we specified general distributive justice principles in light of relevant empirical patterns in the biodiversity field and combined them in subsequent steps that underscore countries’ common but different responsibilities.

In summary, starting from the intuitive notion that those who cause harm ought to correct it, their disproportionate contribution to biodiversity loss at home and abroad implies that the high‐income countries in particular ought to bear a major share of the costs of global biodiversity action. However, the contributor pays principle encounters difficulties with regard to past biodiversity overexploitation and situations where basic livelihoods are at stake. These gaps can partially be filled by the beneficiary pays principle, according to which the higher income countries ought to bear the main share of the global costs for biodiversity action insofar as they have benefited from past biodiversity exploitation. Because not all past biodiversity exploitation has led to lasting benefits and because the beneficiary pays principle does not resolve concerns over overly high burdens on poor countries, a further complementary principle is needed. The fundamental conviction that at least a decent livelihood should be within reach for the residents of all countries suggests allocating all remaining costs of biodiversity action to countries that can be considered able to pay and hence largely exempting lower income countries from any costs. Each principle thus addresses different aspects of the distributive justice challenges faced by global biodiversity policies, but they complement one another in supporting a shared call to action: All countries share a common responsibility to undertake action to protect and recover biodiversity on their territories (i.e., in situ where it is most needed to safeguard the web of life) and to contribute to the best of their capacities. However, given current patterns of causation of biodiversity harm, benefits from past overuse, and widespread poverty, responsibility is highly differentiated and rests largely with the rich industrialized countries. This requires particularly strong practical action, including reducing pollution (target 7) and overexploitation of agricultural land, waters, and forests (target 10) and generally limiting their consumption levels (target 16) and lending financial and technical support to lower income countries.

## 4 INSTITUTIONAL EMBEDDING OF THE NORMATIVE GUIDANCE

There are 2 main institutional mechanisms at the disposal of policy makers and practitioners for a just implementation of the GBF: mechanisms for funding and mechanisms for planning, monitoring, reporting, and review (PMRR). Both are evolving, and we here offer a tentative assessment from a distributive justice perspective. International funding is crucial for a just implementation of the GBF. However, the biodiversity funding gap, globally and in the Global South, has so far been insufficiently addressed. Despite a generic statement of differentiated resource mobilization requirements in the convention (article 20), the GBF's quantified resource mobilization target is the first in the history of the CBD. Its long‐term goal D for 2050 aims for “progressively closing the biodiversity finance gap of $700 billion per year.” As the key interim milestone, target 19 aims to increase overall biodiversity finance to “at least $200 billion per year by 2030” and to increase biodiversity‐related transfers from high‐income to low‐income countries, in particular the least developed and small island developing states, “to at least $20 billion per year by 2025, and to at least $30 billion per year by 2030.”

Yet, valuable time for protecting and restoring ecosystems is lost as the GBF aims to close the biodiversity funding gap but not until 2050. Countries of the Global South moreover believe the figure of US$30 billion in annual international biodiversity transfers by 2030 is insufficient (Abulu & Ghosh, 2022). Although global biodiversity finance is currently US$208 billion annually (Bromley, 2024, p. 16), the GBF's target for 2030 is being met mostly through government spending in the Global North (Bromley, 2024; OECD, 2024). In contrast, the latest available figures suggest that in 2022, total biodiversity‐specific development finance, including private contributions, was US$15.4 billion (OECD, 2024)—20% below the GBF target for 2025. With recent and projected near‐term future cuts in overall official development assistance (OECD, 2025), the effects for biodiversity remain to be seen. Finally, international biodiversity aid flows favor middle‐income over low‐income and least developed countries (OECD, 2024). In sum, the world's poorest countries remain severely underfunded, and an uneven allocation of globally available funds prevails, violating principles of global distributive justice and CBDR.

Besides the mobilization of resources, a reform of the allocation system is thus a pertinent matter of political debate. Even though the largest proportion of international biodiversity funding flows through bilateral channels, the CBD's current multilateral financial mechanism, the Global Environment Facility (GEF), has for long been at the center of attention at COPs (Landry et al., 2024). It is contested by countries of the Global South due to its control by wealthy donor countries and its burdensome bureaucratic procedures that make accessing funds particularly challenging for the poorest countries (ENB, 2025; Landry et al., 2024). In contrast, countries of the Global North defend the GEF as efficient and cost‐effective, and they have warned against the fragmentation of funding mechanisms (ENB, 2025). A compromise decision was made at COP 15 to establish a dedicated Global Biodiversity Framework Fund (GBFF) within the GEF “open to contribution from all sources” (UN, 2022b). It thereby casts the net wider than the GEF, which relies on finance from just 40 donors. The new GBFF was launched in August 2023 (GEF, 2023) and is expected to play a key role in funding GBF implementation (Li et al., 2023). However, low‐income countries still criticized the embedding of the GBFF in the GEF, and at COP 16 they pushed for the establishment of a new financial architecture under the authority of the COP that would give them greater leverage over the allocation of funds (ENB, 2024). Delegates ultimately agreed on a roadmap for deciding on the reform of the CBD's financial mechanisms over subsequent COPs until 2030, potentially leading to the establishment of a new financial entity (UN, 2024b, articles 19 and 22). Given the deep polarization around the issue, many delegates considered this decision ground breaking, but it might lead to difficult negotiations at upcoming COPs (ENB, 2025).

A further key institutional innovation of recent COPs is the strengthening of accountability mechanisms—long overdue given the CBD's weak implementation, often attributed to the absence of compliance mechanisms, including a lack of review (Miller Smallwood et al., 2022). Although the CBD requires all parties to report on their activities (article 26), the lack of standardized content and format has hindered systematic global reviews of the implementation of the convention and its COP decisions. At COP 16, parties adopted a mechanism for PMRR (UN, 2024a) along with a monitoring framework featuring indicators (UN, 2024c). The PMRR introduces a standardized reporting template and a global review and stocktaking process to assess GBF implementation and enable future COPs to recommend actions addressing challenges in the collective progress toward the GBF goals and targets (UN, 2024a, articles 7 and 8). Several observers described the adoption of the PMRR and monitoring framework as a milestone toward greater transparency and accountability for the implementation of the GBF and CBD more broadly (Affinito et al., 2025; ENB, 2025). However, challenges persist related to the comprehensiveness of indicators (ENB, 2025) and the capacity of all parties to report on them. Gaps in data thus will be likely, underscoring the need to improve national monitoring systems over time and to provide financial support for monitoring (Affinito et al., 2025).

Although the PMRR and monitoring framework decisions aim for a global review of progress, thus aggregating national data, we suggest that national data should also be used for tracking the activities of individual parties to assess countries’ alignment with global distributive justice principles. Even if this goes beyond the COPs’ mandate, independent observers—such as academics and nongovernmental organizations—can analyze whether, for example, high‐income countries are reducing polluting activities domestically and abroad, in line with what would be their just contribution to GBF target 7.

Altogether, the current institutional innovations under the CBD and GBF present a significant opportunity to make global biodiversity policies more just. However, there are limits to the effects of institutional reform. As highlighted by the European Union at COP 16, new funding structures do not necessarily translate into new funds (ENB, 2024). Similarly, although the enhanced monitoring mechanism aims to improve accountability, it will still largely depend on countries’ willingness to uphold their commitments—especially because it lacks strong sanctions and enforcement, which are difficult to implement in global policies. Ultimately, what is needed for a just implementation of the GBF in the short time remaining until 2030 is political will.

## Supporting information



Additional supporting information may be found in the online version of the article at the publisher's website.
