# Supplementary material for: Implications of global distributive justice principles for implementation of the Kunming‐Montreal Global Biodiversity Framework
Source: Conserv Biol. 2025 Oct 30;40(1):e70167. doi: 10.1111/cobi.70167 (PMC12856805; doi:10.1111/cobi.70167)
Supplement: Supplementary file 1 — Additional supporting information may be found in the online version of the article at the publisher's website. [file COBI-40-e70167-s001.docx]

**Appendix 1 – List of all GBF Targets**

[TARGET 1](https://www.cbd.int/gbf/targets/1): Plan and Manage all Areas To Reduce Biodiversity Loss

[TARGET 2](https://www.cbd.int/gbf/targets/2/): Restore 30% of all Degraded Ecosystems

[TARGET 3](https://www.cbd.int/gbf/targets/3/): Conserve 30% of Land, Waters and Seas

[TARGET 4](https://www.cbd.int/gbf/targets/4/): Halt Species Extinction, Protect Genetic Diversity, and Manage Human-Wildlife Conflicts

[TARGET 5](https://www.cbd.int/gbf/targets/5/): Ensure Sustainable, Safe and Legal Harvesting and Trade of Wild Species

[TARGET 6](https://www.cbd.int/gbf/targets/6/): Reduce the Introduction of Invasive Alien Species by 50% and Minimize Their Impact

[TARGET 7](https://www.cbd.int/gbf/targets/7/): Reduce Pollution to Levels That Are Not Harmful to Biodiversity

[TARGET 8:](https://www.cbd.int/gbf/targets/8/)Minimize the Impacts of Climate Change on Biodiversity and Build Resilience

[TARGET 9](https://www.cbd.int/gbf/targets/9/): Manage Wild Species Sustainably To Benefit People

[TARGET 10](https://www.cbd.int/gbf/targets/10/): Enhance Biodiversity and Sustainability in Agriculture, Aquaculture, Fisheries, and Forestry

[TARGET 11](https://www.cbd.int/gbf/targets/11/): Restore, Maintain and Enhance Nature’s Contributions to People

[TARGET 12: Enhance Green Spaces and Urban Planning for Human Well-Being and Biodiversity](https://www.cbd.int/gbf/targets/12/)

[TARGET 13](https://www.cbd.int/gbf/targets/13/): Increase the Sharing of Benefits From Genetic Resources, Digital Sequence Information and Traditional Knowledge

[TARGET 14](https://www.cbd.int/gbf/targets/14/): Integrate Biodiversity in Decision-Making at Every Level

[TARGET 15](https://www.cbd.int/gbf/targets/15/): Businesses Assess, Disclose and Reduce Biodiversity-Related Risks and Negative Impacts

[TARGET 16](https://www.cbd.int/gbf/targets/16/): Enable Sustainable Consumption Choices To Reduce Waste and Overconsumption

[TARGET 17](https://www.cbd.int/gbf/targets/17/): Strengthen Biosafety and Distribute the Benefits of Biotechnology

[TARGET 18](https://www.cbd.int/gbf/targets/18/): Reduce Harmful Incentives by at Least $500 Billion per Year, and Scale Up Positive Incentives for Biodiversity

[TARGET 19](https://www.cbd.int/gbf/targets/19/): Mobilize $200 Billion per Year for Biodiversity From all Sources, Including $30 Billion Through International Finance

[TARGET 20: Strengthen Capacity-Building, Technology Transfer, and Scientific and Technical Cooperation for Biodiversity](https://www.cbd.int/gbf/targets/20/)

[TARGET 21](https://www.cbd.int/gbf/targets/21/): Ensure That Knowledge Is Available and Accessible To Guide Biodiversity Action

[TARGET 22](https://www.cbd.int/gbf/targets/22/): Ensure Participation in Decision-Making and Access to Justice and Information Related to Biodiversity for all

[TARGET 23](https://www.cbd.int/gbf/targets/23/): Ensure Gender Equality and a Gender-Responsive Approach for Biodiversity Action

**Appendix 2 – Full text of targets selected for analysis in this article**

Target 2: *Ensure that by 2030 at least 30 per cent of areas of degraded terrestrial, inland water, and coastal and marine ecosystems are under effective restoration, in order to enhance biodiversity and ecosystem functions and services, ecological integrity and connectivity.*

Target 3: *Ensure and enable that by 2030 at least 30 per cent of terrestrial, inland water, and of coastal and marine areas, especially areas of particular importance for biodiversity and ecosystem functions and services, are effectively conserved and managed through ecologically representative, well-connected and equitably governed systems of protected areas and other effective area-based conservation measures, recognizing indigenous and traditional territories where applicable, and integrated into wider landscapes, seascapes and the ocean, while ensuring that any sustainable use, where appropriate in such areas, is fully consistent with conservation outcomes, recognizing and respecting the rights of indigenous peoples and local communities, including over their traditional territories*.

Target 7: *Reduce pollution risks and the negative impact of pollution from all sources, by 2030, to levels that are not harmful to biodiversity and ecosystem functions and services, considering cumulative effects, including: reducing excess nutrients lost to the environment by at least half including through more efficient nutrient cycling and use; reducing the overall risk from pesticides and highly hazardous chemicals by at least half including through integrated pest management, based on science, taking into account food security and livelihoods; and also preventing, reducing, and working towards eliminating plastic pollution.*

Target 10: *Ensure that areas under agriculture, aquaculture, fisheries and forestry are managed sustainably, in particular through the sustainable use of biodiversity, including through a substantial increase of the application of biodiversity friendly practices, such as sustainable intensification, agroecological and other innovative approaches contributing to the resilience and long-term efficiency and productivity of these production systems and to food security, conserving and restoring biodiversity and maintaining nature’s contributions to people, including ecosystem functions and services.*

Target 11: *Restore, maintain and enhance nature’s contributions to people, including ecosystem functions and services, such as regulation of air, water, and climate, soil health, pollination and reduction of disease risk, as well as protection from natural hazards and disasters, through nature-based solutions and/or ecosystem-based approaches for the benefit of all people and nature.*

Target 16: *Ensure* *that people are encouraged and enabled to make sustainable consumption choices including by establishing supportive policy, legislative or regulatory frameworks, improving education and access to relevant and accurate information and alternatives, and by 2030, reduce the global footprint of consumption in an equitable manner, including through halving global food waste, significantly reducing overconsumption and substantially reducing waste generation, in order for all people to live well in harmony with Mother Earth.*
